# Analysing the Motions of Spray Droplets on a Cow’s Surface to Relieve Heat Stress

**DOI:** 10.1038/s41598-018-38354-0

**Published:** 2019-02-14

**Authors:** Guangzhi Li, Zonglun Wang, Zhengxiang Shi, Tao Ding, Qian He, Shuai Hong

**Affiliations:** 1 College of Water Resources and Civil Engineering, China Agricultural University, Beijing, 100083 China; 2 Beijing Engineering Research Center of Safety and Energy Saving Technology for Water Supply Network System, Beijing, 100083 China; 3 Key Laboratory of Agricultural Engineering in Structure and Environment, Beijing, 100083 China

## Abstract

Exploring the behaviour of sprayed water droplets on dairy cow hair during the spraying process is of great significance to improve the effects of this process on cooling a dairy cow’s body. In this paper, we use a high-speed camera to examine the sprayed droplets of different diameters and then analyse the experimental results. The results show that the movements of sprayed droplets on the simulated dairy cow (**SDC**) surface can be divided into four categories: random scattering, aggregation, multiple deformations and flow slipping. Sprayed droplets with diameters of 0.56 *mm* and 0.8 *mm* exhibit more frequent random scattering than do other droplets. However, this behaviour is unfavourable for cooling the dairy cow body. By analysing the dimensionless parameter *B*, we find that sprayed droplets with a diameter of 1.1 *mm*, which have a higher frequency of aggregation, is not conducive for cooling the dairy cow body. However, multiple deformations can contribute to the cooling process of a SDC. By analysing the relationship between *We* and *γ*, we can find the range of *We* and *γ* in which the behaviour of random scattering and multiple deformations may appear more frequently. The results show that sprayed droplets with diameters of 0.8 *mm*–1.0 *mm* exhibit multiple deformations more frequently, which is beneficial for the cooling process of a SDC.

## Introduction

Spray cooling is an important method for alleviating the heat stress of dairy cows^1–3^ in the summer, which can effectively reduce their surface temperature, prevent the physiological discomfort caused by heat stress and ensure milk production^4^. The mechanism of spray cooling is the heat evaporation of droplets on the cow surface. During the evaporation process, the droplets must exhibit certain movement phenomena. Through analysis, we can obtain the relationship between the droplets’ movement and their sizes and then study the movement characteristics of sprayed droplets and the mechanism of spray evaporation.

To date, much work has been conducted on the crushing characteristics of a droplet hitting a wall^5–7^. Researchers have studied the spreading movement of a single droplet through experimental phenomena and numerical simulations^8–10^. Rioboo^11^ *et al*. studied the relationship between the spreading regulation and the droplet size, striking velocity, and wall material when droplets hit different dry solid walls by an experimental method. Shen^12^ pointed out the contact time of impact water droplets on superhydrophobic surfaces directly reflects the extent of thermal and energy conversions between the water droplet and the surface. Yahua Liu^13^ show how superhydrophobic surfaces patterned with lattices of submillimetre-scale posts decorated with nano-textures can generate a counter-intuitive bouncing regime. Gauthier Anaïs^14^ propose a quantitative analysis of the reduction of contact time and thus to understand how and why macrotextures can control the dynamical properties of bouncing drops. Patricia^15^ found that variations in temperature do not influence the wettability and mobility of the water droplets. Patricia found that variations in temperature do not influence the wettability. There’s a complete bouncing when a droplet impacting on a superhydrophobic surface, and the impacting process usually consists of spreading and retracting stages^16^. Negeed^17^ used high-speed camera record droplet behavior in collision with hot surfaces, and used empirical relationships described the characteristics of droplets impinging on heated hydrophilic surfaces. Haibao Hu^18^ *et al*. also used a high-speed camera to study the freezing process when water droplets hit a smooth wall at a low temperature. Yunchao SONG^19–21^ *et al*. used the numerical simulation method to realize the numerical solution of the spreading movement when droplets hit a wall. In addition, they analysed the dimensionless parameters in the moving process.

The studies mentioned above mainly investigated a hydrophobic solid wall for single droplets. However, there are very few studies of sprayed droplets on a water-infiltrated cow hair surface using either experimental collection or numerical simulation. This paper considers the effect of water droplets in the spray cooling process. The statistical average method is adopted because the observation of a single droplet is limited in production and application. By using a high-speed photographing system, we collect records of the spray process over a given period of time and then observe, describe and analyse the video to explore the movement of sprayed droplets and the relationship between the movement and the characteristics of water droplets. This can also provide a theoretical basis for further study on the morphology of water droplets on cow surface and the heat transfer mechanism between the body and the surrounding environment.

## Experimental Method and Setup

### Experimental device

**Figure 1:**
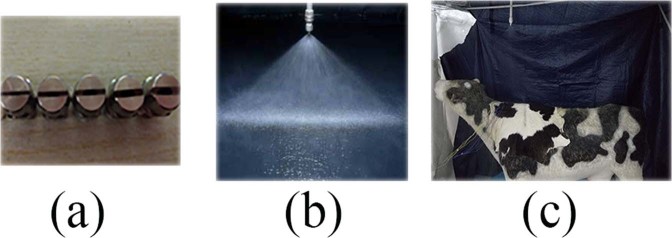
Sprayer and cow model: The shapes of the nozzles are shown in (**a**), and the spray pattern is shown in (**b**).

The experiment was conducted in the Laboratory of Water Resources and Civil Engineering, China Agricultural University. Shuai Hong^22^ and group members tested different Japanese pool standard fan-shaped nozzles. According to the spray pressure, they finally chose the 9030, 9060, 9080, 90100, and 90120 model nozzles. The shapes of the nozzles are shown in Fig. 1(a), and the spray pattern is shown in Fig. 1(b).

The 1:1 SDC model was used as the research object shown in picture 1 (c), and the SDC has a heating tungsten wire (temperature ranging from 0 °C to 100 °C, with a resolution of 0.1 °C) to guarantee the surface temperature. And because the cows are warm-blooded animals, the body temperature of the dairy cow model was kept at 39 °C by the heating tungsten wire during the spraying experiment, while ambient temperature is 35 °C^22^. The material of the SDC skin is silicone with dairy cow hair to ensure the accuracy of the surface characteristics of dairy cow. The installation height of the nozzle was 2.2 *m*, which was 70 *cm* above the SDC back with reference to a domestic dairy farm. The spray pressures were 0.15 *MPa*, 0.20 *MPa* and 0.25 *MPa*. A precise pressure gauge was used to collect data. The neck area is the sensitive part on the cow for easing the heat stress^23^, so this test mainly shot the neck area using the high-speed camera.

The video of sprayed droplets on the SDC surface was recorded by a United States *VRI* Phantom high-speed camera. The final shooting parameters are as follows: resolution: 1280 × 800, exposure time: 398.8 *μs*, recording time for a single picture in one video: 526.32 *μs*, number of pictures: 5500, total length of one video: 2.9 ± 0.1 *s* (thus, each time that we shot for 2.9 *s*, the error was 0.1 *s*; within the 2.9 *s* shooting time, we could acquire approximately 5000 images).

**Figure 2:**
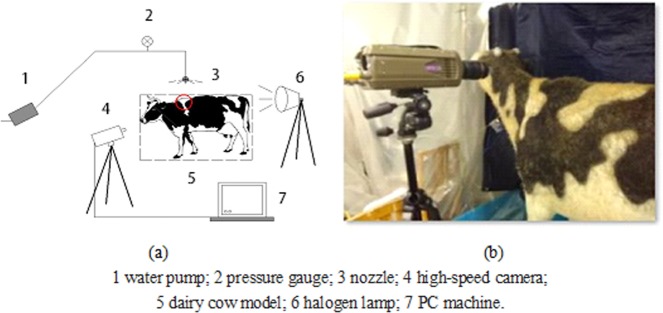
Spray test equipment: The placement of the experimental instruments is shown in Fig. 2.

A 220 *V* halogen lamp was used as a light source to assist the shooting with the high-speed camera. Because of the high speed of heat production, the single working time of halogen lamp is controlled within 1 minute, with an interval of 2–3 minutes to guarantee the lighting effect and life expectancy. The final placement of the experimental instruments is shown in Fig. 2.

### Experiment method

#### Test shoot

After all pieces of equipment were installed, we opened the water valve and adjusted it to acquire a pressure of 0.15 *MPa*, according to the pressure gauge, and then waited for 10 *s* to acquire a stable water droplet. Subsequently, we opened the halogen lamp, adjusted the focal length, and made the image appear on the previously set location. We then clicked the button that controlled the high-speed camera on the computer to start shooting. After the ending single appeared, we turned off the halogen lamp and the water valve, saved the recorded video, and finished the shooting process. We continued to shoot the spray water with the same nozzle under pressures of 0.20 *MPa* and 0.25 *MPa* respectively. Then, we changed the nozzle, repeated the operation above, and saved the spray videos of different nozzles with different pressures.

#### Analysing the principles

**Figure 3:**
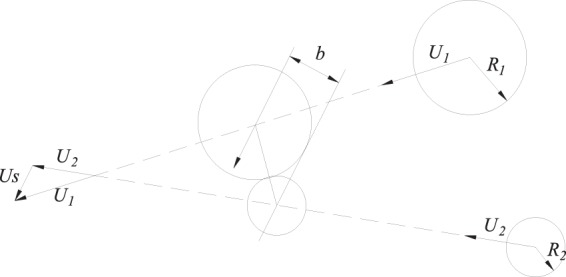
Dimensionless parameter B: *R1* and *R2* are the radii of two moving droplets, respectively, measured in *m*; and *b* is the space parameter of droplets, which is the projection length of the centre line of the collision droplets in the direction of their relative velocity vector, as shown in Fig. 3.

The experimental analysis included analysing the experimental videos and the relationship between the average droplet sizes and the characteristic parameters. We choose the dimensionless parameter *B*, ratio of droplet radius *γ* and Weber number *We* for analysis. *B* is a parameter that describes the relative motion of two droplets, and *We* is a common parameter that affects the motion of droplets^24,25^. By analysing the relationship between *We* and *γ*, we can study the relationship between the droplets’ movements on the cow surface and their average particle sizes. The dimensionless parameter *B* is expressed as follows:

1 $$B=\frac{b}{{R}_{1}+{R}_{2}}$$

where *R*_*1*_ and *R*_*2*_ are the radius of two moving droplets, respectively, measured in *m*; and *b* is the space parameter of droplets, which is the projection length of the centre line of the collision droplets in the direction of their relative velocity vector, as shown in Fig. 3.

The average Weber number of the droplet is the ratio of the inertial force to their surface tension and is commonly expressed as

2 $$We=\frac{\rho ({r}_{1}+{r}_{2}){v}^{2}}{\sigma }$$

where *r*_*1*_ is the initial mean radius of the sprayed water droplet, r2 is the radius of the water droplet at the initial time when a certain droplet behaviour appears, and *ρ* is the density of water, *kg*/*m3*. *v* is the relative velocity of two droplets, and *m*/*s*. *σ* is the coefficient of surface tension, *N*/*m*.

The characteristics of experimental water droplets are^26^ shown in Table 1:

**Table 1:** Characteristic parameters of experimental liquid.

Experimental liquid	*ρ*/*kg*.*m*^−3^	*σ*/*N*.*m*^−1^
water	1000	0.072

The droplet radius ratio γ is

3 $$\gamma =\frac{{r}_{2}}{{r}_{1}}$$

where *r*_*1*_ is the initial mean radius of the sprayed water droplets and r2 is the radius of the water droplets at the initial time when a certain droplet behaviour appears.

The initial average particle size *r*_*1*_ of the sprayed water droplet is specified in Section 3.2.1. We use the SDC body as a reference to determine the parameter *r*_*2*_. The actual SDC diameter is *d*, and the diameter of the SDC in the high-speed photographic image is *d*_*0*_. The actual diameter of the water droplet is *D* (*D* = *2r*_*2*_), and the diameter of the droplet in the high-speed photographic image is *D*_*0*_. Then, *d*, *d*_*0*_ and *D*_*0*_ can be measured by a Vernier caliper and the image size of the high-speed photograph. The Vernier caliper is an industrial caliper with a range of 150 *mm* and a precision of 20 *μm* to meet the test requirement. The scale conversion equation is as follows:

4 $$\frac{d}{{d}_{0}}=\frac{D}{{D}_{0}}$$

Then, the actual size of the water droplet can be expressed as *r*_*2*_ = *D*/*2*.

## Results and Analysis

### Movement of water droplets

By analysing the experimental videos, the behaviour of water droplets on the SDC surface can be divided into four categories: random scattering, fusion aggregation, multiple deformation and flow slipping.

#### Random scattering

**Figure 4:**
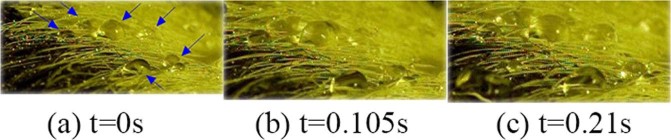
Random Scattering Behaviour: A variable number of these new droplets scatter randomly and do not continue to gather with surrounding water droplets or slide along.

After the spray droplets drip on the SDC surface, some water droplets will gather with subsequent ones to form a new droplet that has a certain size and shape. A variable number of these new droplets scatter randomly and do not continue to gather with surrounding water droplets or slide along the surface of the SDC. These droplets semi-statically stay at the place where they are formed, as Fig. 4 shows.

Setting the first picture as the initial time (the following behaviour also use the same analysis method), we can see that the dropped water shows no difference but only shows a slight location change in the next 0.21 s, even though the spraying process is continuing. We call this behaviour “random scattering”.

#### Fusion aggregation

**Figure 5:**
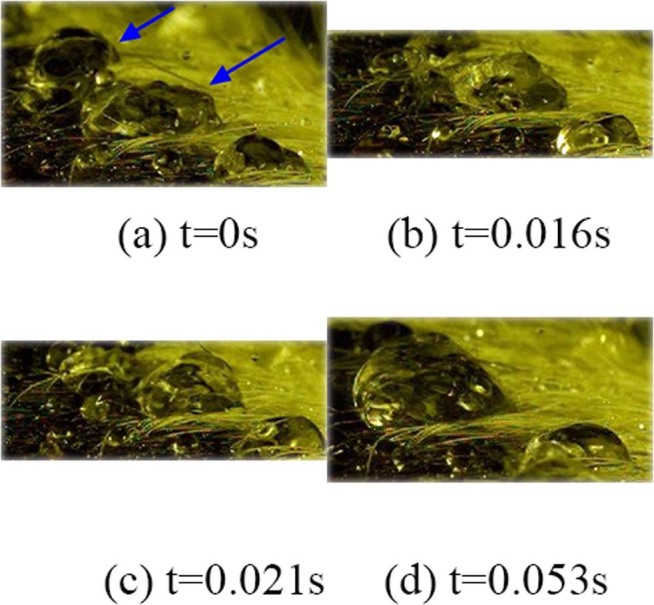
Aggregation Behaviour: Because of the influence of shaking, the gathered droplets will aggregate again with the surrounding droplets. With the help of subsequent water droplets, the gathered droplets will also aggregate with the droplets at a distance.

Because of the influence of shaking, the gathered droplets will aggregate again with the surrounding droplets. With the help of subsequent water droplets, the gathered droplets will also aggregate with the droplets at a distance, as shown in Fig. 5.

The pictures show the process of droplet aggregation within 0.053 s. From the pictures, we can find that at 0 s, the marked two water droplets do not contact and that a gap exists between them. After a certain period of time, at 0.016 s, the two water droplets begin to contact, and there exists a trend of mutual integration. At 0.021 s, the two water droplets are slightly gentle in the process of aggregation, which is due to the vibration offset between the water droplets, but this vibration offset does not appear in each aggregation process. Although the combination of two water droplets appears to be gentle at the last moment, this does not affect the tendency for them to gather. At 0.053 second, the two water droplets eventually fully converge together’ that is, the fusion process is completed.

#### Multiple deformations

Multiple deformations mean that the shapes of the gathered droplets are influenced by the continuously dripping water. Multiple deformations are the most abundant spray water droplet behaviour and are irregular and unpredictable. For convenience, multiple deformations can be re-classified as broken deformation and non-crushing deformation according to whether the water droplets are broken. Non-crushing deformation includes spherical surges and vortex surges, and broken deformation includes segmental broken and exploded broken.

Non-crushing deformation: Non-crushing surge refers mainly to the surge behaviour in which the shapes of the gathered water droplets continue to change under the influence of subsequent water droplets. It is characterized by the observation that only a small amount of the water droplets of the original body (the surging water droplets) is splashed out and the original body itself is not broken throughout the changing process.

**Figure 6:**
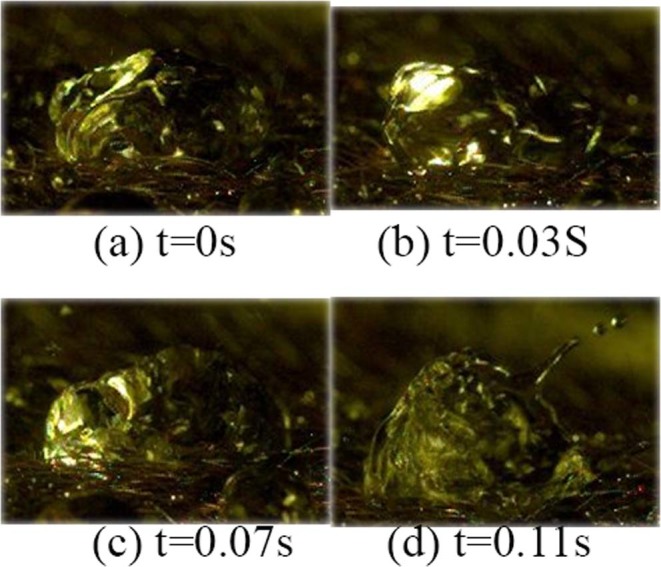
Spherical Deformation Behaviour: Spherical surge is when the shape of the droplet is spherical when a surge occurs. It is characterized by the observation that only a small amount of water droplets is splashed out and the original body is not broken.
  It can be seen from the pictures that at 0 s, the spherical surge behaviour is revealed. The water droplets are no longer smoothly spherical, protrusions appear on the top, and the body begins to contract. At 0.03 s, the water droplets change more sharply than at the previous time. Shrinkage and protrusion are more obvious, but the locations are different, which is the irregular deformation behaviour that is observed when surge occurs. At 0.07 s, small water droplets in the outer layer of the original body begin to split out due to the violent surge and further development. At 0.11 s, as the surge continues, some of the separated small water droplets of the original body are splashed out; the others are re-involved in the following surge process.
- Vortex surge
  Vortex surge is when the shape of the droplet is a swirl when surge occurs. It is characterized by the observation that the middle of the droplets is depressed and other parts are tilted, thus forming a vortex with a certain thickness that is short and unstable, as shown in Fig. 7.

- Spherical surge
  Spherical surge is when the shape of the droplet is spherical when a surge occurs. It is characterized by the observation that only a small amount of water droplets is splashed out and the original body is not broken, as shown in Fig. 6.

**Figure 7:**
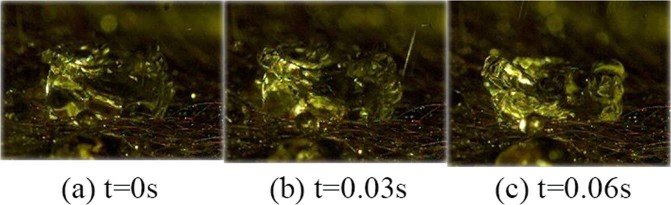
Vortex Deformation Behaviour: Vortex surge is when the shape of the droplet is a swirl when surge occurs. It is characterized by the observation that the middle of the droplets is depressed and other parts are tilted, thus forming a vortex with a certain thickness that is short and unstable.
  It can be seen from the pictures above that the top surrounding parts of the water droplets are raised and that the middle parts are drum-shaped. The tilt gradually becomes thicker as the surge continues, and the “scroll” tends to be reduced.
  The vortex surge and spherical surge both belong to the type of non-crushing deformation and have the relevant characteristics. However, the two behaviour are relative, not antagonistic, and there is no clear boundary between these behaviour. Spherical surge can appear solely, it can appear after the vortex surge, and these two behaviour can even appear alternately.
  Broken deformation: Broken deformation can be divided into segmental broken and exploded broken. The main feature is that the original water droplets appear in different degrees.
- Segmental broken
  Segmental broken refers to water droplets that have been dropped on the ox hair and gathered to form a certain size of the spray water droplets under the influence of subsequent water droplets, thus making the original water droplets break partly. It can also be called a broken edge. This is shown in Fig. 8.

**Figure 8:**
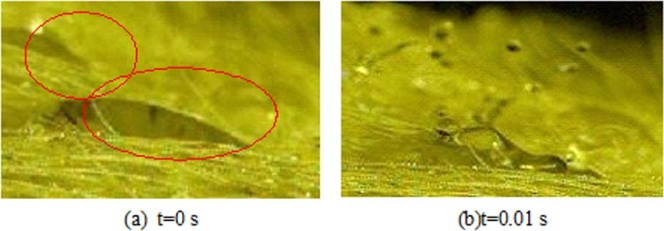
Segmental Broken Behaviour: Segmental broken refers to water droplets that have been dropped on the ox hair and gathered to form a certain size of the spray water droplets under the influence of subsequent water droplets, thus making the original water droplets break partly.
  It can be seen from the figure that at 0 s, the original body (the fragmented water droplets) gathers to form a droplet with a single shape and size. At 0.01 s, due to the subsequent water droplets, the original right side of the edge breaks out, and small water droplets splash, but the left part remains intact.
- Exploded broken

**Figure 9:**
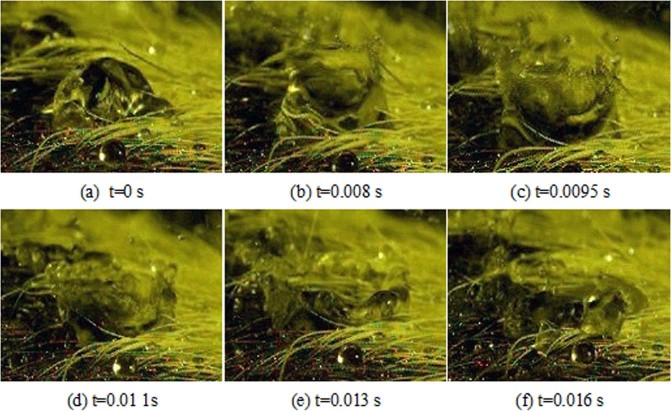
Exploded Broken Behaviour: Exploded broken refers to when the water droplets which have been dripping on the ox hair gather to form a droplet with a certain size, thus making the original droplet break to a significant degree under the influence of subsequent water droplets.

Exploded broken refers to when the water droplets which have been dripping on the ox hair gather to form a droplet with a certain size, thus making the original droplet break to a significant degree under the influence of subsequent water droplets. The whole process is short and violent, as shown in Fig. 9.

From the pictures above, we can find that at 0 s, the spray water droplets that have been dripped and gathered present a common form. At 0.008 s, the water droplets begin to break, and when the water droplets break, the middle part depresses, while the surrounding part shows different degrees of protrusion. At 0.0095 s, the degree of crushing increases, and the middle depression part increases and deepens further. The surrounding part of the protrusion rises to almost the maximum height, and only one thin layer of water remains for all of the water droplets. At 0.011 s, the surrounding protrusions become shorter and tend to fall, and the surrounding water film becomes thicker than before owing to this tendency. At 0.013 s, the protruding part of the broken water droplets continue to become thicker and shorter than the previous stage, while the middle depression part becomes wider; at this point, the water droplets are already spreading. At 0.016 s, the surrounding part of the protruding part has fallen. The original body has spread on the surface of the hair, and only one circle of water droplets is left discontinuously. At this point, the broken process is over, and the whole process lasts for 0.016 second, which shows the short duration of the broken process.

The exploded broken is similar to the crown of a single droplet hitting a specific wall^27^. However, the difference in the contents, the environment and the form of the experiment contribute to some of the discrepancies in the behaviour, which requires further experiment and study. The exploded broken of the experiment already shows that the behaviour of sprayed water droplets have many similarities to that observed by SONG Yunchao and other researchers. For example, a thin layer of water forms when Rioboo^11^ observed the droplet impact process.

Segmental broken and exploded broken are relative but not opposing, and no definite boundaries exist between them. They both have characteristics of broken deformation, such as transience, which leads to the different broken degrees of the original body.

#### Flow slipping behaviour

Flow slipping, as the name implies, refers to when the water droplets overcome friction to flow and slip along the irregular path after they are gathered. However, there is a slight difference between the droplets flowing on the ox hair and those flowing on the floor. At the very beginning, the water droplets will not flow alone due to their small sizes and friction; only when they are gathered to a certain extent can they flow. When flow occurs, it tends to form a small stream of water or a separate water droplet rather than a continuous or a regular stream. From the video, the flow behaviour is less common than the other three types of behaviour.

### Statistical analysis of the behaviour and the average particle size of sprayed water droplets

#### Determination of the average particle size of sprayed water droplets

To discuss the behaviour of sprayed water droplets more intuitively, we classify the average particle size of the water droplets in the experiment. Shuai Hong^22,28^ tested the sizes of sprayed water droplets and acquired statistics under different working pressures, as shown in the following Table 2.

**Table 2:** Particle size classification of sprayed droplets.

Nozzle	Working pressure (MPa)	Average particle size(mm)	Droplet speed (m/s)	Kinetic energy 10^−6^ (J)
9030	0.15	0.57	3.38	7.53
	0.20	0.56		
	0.25	0.55		
r1		0.56		
9060	0.15	0.81	3.44	23.36
	0.20	0.78		
	0.25	0.79		
r1		0.80		
9080	0.15	0.91	3.69	39.58
	0.20	0.89		
	0.25	0.88		
r1		0.90		
	0.15	1.04		
90100	0.20	1.03	3.92	62.09
	0.25	0.99		
r1		1.00		
90120	0.15	1.12	4.20	98.35
	0.20	1.11		
	0.25	1.11		
r1		1.10		

#### Statistical analysis of droplets’ behaviour

In the analysis procedure, the average particle sizes of the sprayed droplets of different nozzles are divided and analysed. For the spray videos with the same particle size, the video will start playing randomly. Within a fixed observation period *T*. The number of all the motion phenomena of the spray water droplets occur is recorded as *N*, and the number of any one motion phenomenon is recorded as *n*; thus, the frequency (*p* = *n*/*N*) of the movement of water droplets is obtained.

**Figure 10:**
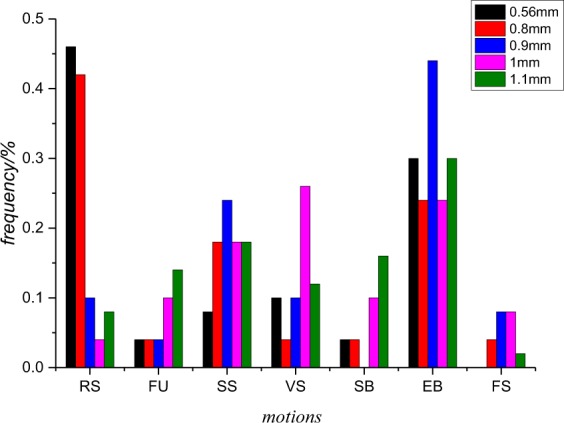
Statistics of spray droplet behaviour: The number of total droplets occurring during the *T* times is denoted as *N*, and the number of total droplets exhibiting a certain specific behaviour during the *T* times is denoted as *n*; thus, the frequency (*p* = *n*/*N*) of the movement of water droplets is obtained.

The frequencies of all behaviour under different particle sizes are analysed, and we can obtain the graph below:

From the Fig. 10 above, we can see that the frequency of random scattering differs greatly as the average particle size changes. Smaller water droplets with average particle sizes of 0.56 mm and 0.80 mm tend to exhibit random scattering. The frequency of multiple deformation under any average particle size is larger than that of the other three types (random scattering (RS), fusion (FU) and flow slipping (FS)), and exploded broken appears the most frequently.

### The relationship between droplets’ movements and characteristic parameters

We use the dimensionless parameter *B* to analyse the average particle sizes and the fusion aggregation behaviour of the sprayed water droplets, and use *We* and *γ* to study the characteristics and regulations of random scattering and multiple deformations of sprayed droplets. The flow slipping behaviour is not discussed because of its short residence time and weak characteristic.

#### The relationship between fusion aggregation and dimensionless parameter *B*

**Figure 11:**
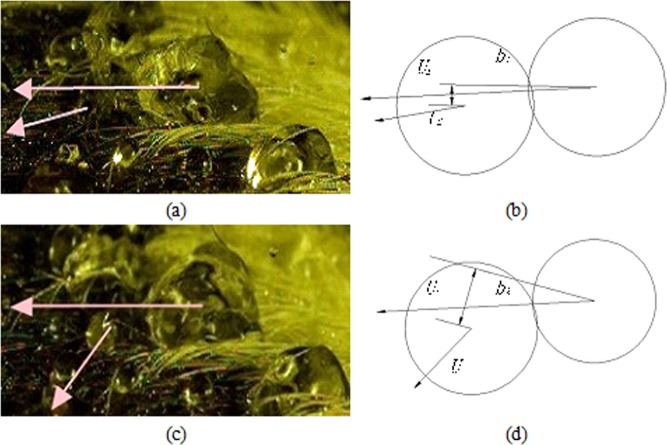
The behaviour of different times and the decomposition of aggregation of spray droplets.

When fusion aggregation occurs, the dimensionless parameter *B* changes significantly due to the random movements of the sprayed droplets. Figure 11 shows that the two gathering droplets show different moving tendencies in different times, which results in the difference in the length of projection “*b*” of their centre line “*a*” in the normal direction of their relative velocity vector. The dimensionless parameter *B1* < *B2* because *b1* < *b2*. The characteristic of dimensionless parameter *B* is that when *B* = 0, the two droplets move along the central line; when *B* = 1, the two droplets move tangentially at the contact point. Picture 11 shows that when *B1* < *B2*, the tendency of two droplets gathering together is much higher than that at the moment *t2*, which shows the randomness and uncertainty of the gathering movement of two droplets.

#### Weber number and droplet radius ratio

**Figure 12:**
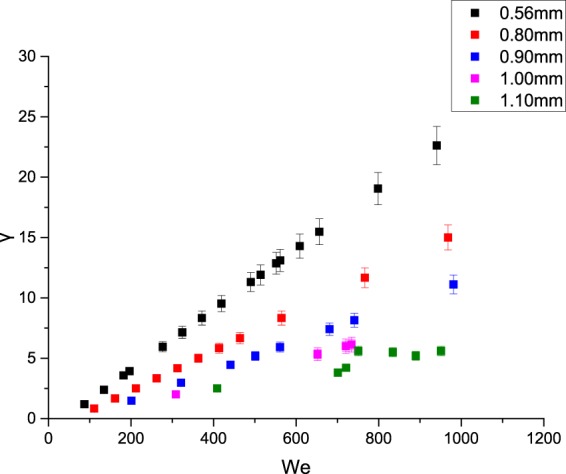
Relationship between *We* and *γ* of the random scattering behavior.

For the random scattering and multiple deformations, statistical analysis finds that the dimensionless parameter *We* and the ratio of two droplets’ radii *γ* have a certain relationship, as shown in the graph below:

Figure 12 shows that when random scattering occurs, *γ* mainly concentrates in the region of 0 to 20, and *We* changes in the range of 100 to 1000; that is, when the inertial force is not obvious, the surface tension can still maintain the morphology of water droplets moving on the surface, in which case the sprayed droplets tend to exhibit random scattering.

**Figure 13:**
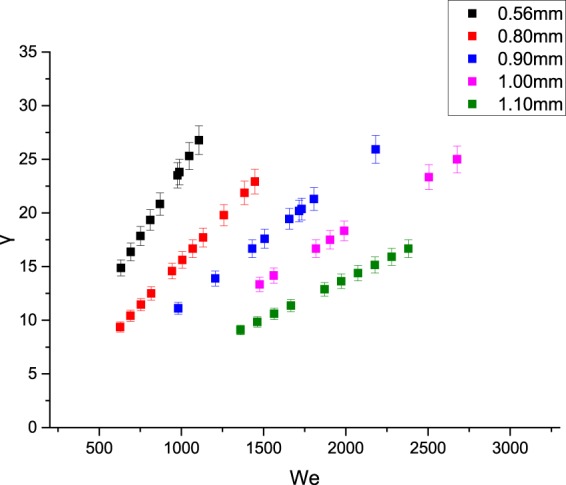
Relationship between *We* and *γ* of the multiple deformations.

Figure 13 shows that when multiple deformations occur, *γ* mainly concentrates in the region of 5 to 30, while *We* changes in the region of 500 to 3000. When *γ* is constant, *We* increases as the sizes of droplets increase, which indicates that the hitting impact is more obvious because of the large kinetic energy of large particle sizes. Thus the inertial force plays a dominant role respect to the surface tension, and the surface tension cannot maintain a “stable form” of the water droplets on the surface, which results in multiple deformations are more likely to occur.

### Analysis of movements of water droplets

In this experiment, we use a high-speed camera to acquire the moving behaviour of water droplets and study the moving characteristics from a microscopic point of view. The best effect of spray cooling is to infiltrate the dairy cow surface without water droplets dripping off and without spraying the breast^29^. Water droplets must form an effective water film (just infiltrating the ox hair) to facilitate heat evaporation while not flowing off the dairy cow surface (not conducive to heat transfer) when the droplets infiltrate the dairy cow. Therefore, it is very important to explore the movements and morphological changes of sprayed droplets on the surface of a dairy cow’s body.

According to Fig. 10, droplets with average sizes of 0.56 mm and 0.80 mm are much more likely to exhibit random scattering among the five different droplet sizes considered. When random scattering occurs, the water droplets hang on the surface of ox hair and cannot infiltrate the dairy cow body, thus hindering the heat transfer. Therefore, small droplets of 0.56 mm and 0.80 mm sizes are not suitable for spray cooling according to random scattering.

The dimensionless parameter *B* between two sprayed droplets is decisive when fusion gathering occurs. If *B* is too small (close to 0), the two droplets tend to move along the centre line to gather into a larger droplet, which hinders the heat transfer of the dairy cow body and is adverse to evaporation. If *B* is too large (close to 1), the two water droplets tend to move tangentially, and there is no obvious aggregation behaviour. The two droplets can still have multiple deformations in the following spraying process and can thus contribute to the spray cooling. As shown in Fig. 10, droplets with an average particle size of 1.10 mm may exhibit such a behaviour more frequently. We do not suggest select droplets with a size of 1.1 mm due to the waste of water and high frequency of gathering behaviour.

Figures 12 and 13 show the relationship between *We* and *γ* when random scattering and multiple deformations occur, based on which we analyse the interaction between the inertia force and the surface tension and their relationship with the ratio of the droplet radius. Further study shows that as *We* increases, the inertia force gradually dominates, which makes the multiple deformations occur more frequently. To conclude, droplets of 0.8 mm to 1.0 mm have a higher frequency of multiple deformations, thus contributing to the spray cooling. The project team also has relevant papers studying the heat transfer of droplets with radius of 0.8 mm to 1.0 mm.

## Conclusion

1. According to the experimental results, the movements of spray droplets can be divided into four categories: random scattering, fusion aggregation, multiple deformations and flow slipping. Multiple deformations can be divided into non-broken deformations and broken deformations.
2. According to the results, droplets of 0.56 mm and 0.80 mm may exhibit random scattering more frequently, but this is adverse to spray cooling. And the droplets of 1.10 mm of high frequency of fusion aggregation are also unfavourable for spray cooling considering the waste of water. So we do not recommend the use of droplets of these sizes.
3. By analysing the dimensionless parameter *B*, we can conclude that fusion aggregation is unfavourable for spray cooling and that multiple deformations contribute to the spray cooling. Considering the waste of water and the high frequency of fusion aggregation, droplets of 1.1 mm are not recommended.
4. By analysing the relationship between *We* and *γ*, we can conclude that when random scattering occurs, *γ* mainly concentrates in the range of 0 to 20, while *We* is in the range of 100 to 1000. When multiple deformations occur, *γ* mainly concentrates in the region of 5 to 30, while *We* changes in the range of 500 to 3000. Droplets with radius of 0.8 mm to 1.00 is conductive to spray cooling which have a higher frequency of exhibiting multiple deformations.

## Data Availability

All data generated and analysed during this study are included in this published article and the datasets generated and analysed during the current study are available from the corresponding author on reasonable request.
